# IFN-γ Plays a Unique Role in Protection against Low Virulent *Trypanosoma cruzi* Strain

**DOI:** 10.1371/journal.pntd.0001598

**Published:** 2012-04-03

**Authors:** Adele A. Rodrigues, Jasson S. S. Saosa, Grace K. da Silva, Flávia A. Martins, Aline A. da Silva, Cecílio P. da Silva Souza Neto, Catarina V. Horta, Dario S. Zamboni, João S. da Silva, Eloisa A. V. Ferro, Claudio V. da Silva

**Affiliations:** 1 Instituto de Ciências Biomédicas, Universidade Federal de Uberlândia, Uberlândia, Minas Gerais, Brazil; 2 Departamento de Bioquímica e Imunologia, Universidade de São Paulo, Ribeirão Preto, São Paulo, Brazil; 3 Universidad Autónoma de Bucaramanga, Bucaramanga, Colômbia; 4 Departamento de Biologia Celular, Universidade de São Paulo, Ribeirão Preto, São Paulo, Brazil; Institut Pasteur, France

## Abstract

**Background:**

*T. cruzi* strains have been divided into six discrete typing units (DTUs) according to their genetic background. These groups are designated *T. cruzi* I to VI. In this context, amastigotes from G strain (*T. cruzi* I) are highly infective *in vitro* and show no parasitemia *in vivo*. Here we aimed to understand why amastigotes from G strain are highly infective *in vitro* and do not contribute for a patent *in vivo* infection.

**Methodology/Principal Findings:**

Our *in vitro* studies demonstrated the first evidence that IFN-γ would be associated to the low virulence of G strain *in vivo*. After intraperitoneal amastigotes inoculation in wild-type and knockout mice for TNF-α, Nod2, Myd88, iNOS, IL-12p40, IL-18, CD4, CD8 and IFN-γ we found that the latter is crucial for controlling infection by G strain amastigotes.

**Conclusions/Significance:**

Our results showed that amastigotes from G strain are highly infective *in vitro* but did not contribute for a patent infection *in vivo* due to its susceptibility to IFN-γ production by host immune cells. These data are useful to understand the mechanisms underlying the contrasting behavior of different *T. cruzi* groups for *in vitro* and *in vivo* infection.

## Author Summary

*Trypanosoma cruzi*, an obligate intracellular protozoan, is the etiological agent of Chagas disease that represents an important public health burden in Latin America. The infection with this parasite can lead to severe complications in cardiac and gastrointestinal tissue depending on the strain of parasite and host genetics. Currently, six genetic groups (*T. cruzi* I to VI) have been identified in this highly genetic and diverse parasite.The majority of published data concerning host immune response has been obtained from studying *T. cruzi* II to VI-infected mice, and the genetic differences between *T. cruzi* II to VI and *T. cruzi* I strains are large. Here we aimed to understand how amastigotes from *T. cruzi* I G strain are highly infective *in vitro* and do not contribute for a patent parasitemia *in vivo*. Our results showed that amastigotes from G strain are highly susceptible to IFN-γ treatment *in vitro* and secretion by immune cells *in vivo*. This information may represent important findings to design novel immune strategies to control pathology that may be caused by different strains in the same host.

## Introduction

Chagas disease is a chronic, systemic, parasitic infection caused by the protozoan *Trypanosoma cruzi*. The disease affects about 8 million people in Latin America, of whom 30–40% either have or will develop cardiomyopathy, digestive megasyndromes, or both [1]. Knowledge of the pathology and immune response to *T. cruzi* infection has been largely obtained from murine models. These models have shown that the innate and adaptive immune responses play an important role in parasite control, depending on the combined action of various cellular types including NK, CD4+ and CD8+ as well as on the production of antibodies by B cells [2]–[5]. Resistance to *T. cruzi* infection has been associated with the production of the pro-inflammatory cytokines IL-12 and IFN-*γ* and with the local production of RANTES, MIP-1*α*, MIP-1*β* and MCP-1. These cytokines activate the production of nitric oxide by macrophages, which is responsible for elimination of the parasite [6]–[9]. TNF-*α* has also been associated with macrophage activation as a secondary signal for nitric oxide production [10]. In contrast, cytokines such as IL-4 and TGF-*β* are associated with parasite susceptibility [11], [12].

*T. cruzi* strains have been divided into six discrete typing units (DTUs) according to their genetic background. These groups are designed *T. cruzi* I to VI [13]. The geographical distribution of these groups indicate that *T. cruzi* II to VI are the main causal agent of Chagas' disease in the southern parts of South America, with *T. cruzi* I only present in the sylvatic cycle [13]–[15]. In contrast, in Colombia, Venezuela, and Central America *T. cruzi* I have been reported as the primary parasite present in human cases [16]–[18].

*T. cruzi* G strain (obtained from Nobuko Yoshida and originally from Mena Barreto), isolated from an opossum in the Amazon region, belongs to genotype I and shows a particular behavior; Metacyclic trypomastigotes from G strain show low infectivity *in vitro* and no *in vivo* parasitemia [19]. This phenotype was attributed to the expression of a glycoprotein GP90, a stage specific negative modulator of cell invasion [20]. Conversely amastigotes from G strain are highly infective *in vitro* [21], [22] but do not sustain a patent infection *in vivo* (data not published), regardless that amastigotes and blood stream trypomastigotes are the main forms encountered during the disease progression. Indeed, the presence of blood stream typomastigotes reflects the full completion of the developmental parasite life cycle program otherwise known to proceed in the most frequent conditions. These results raised questions that remained to be addressed along the past years. How come amastigotes from G strain are highly infective *in vitro* and do not contribute for a patent infection *in vivo*? How would the host respond to the infection that complete abolishes parasitemia? Thus, to gain insight concerning this issue we performed this study that provided us a unique result; IFN-γ production *per se* is sufficient to control infection by G strain amastigotes. It is extensively known the role of IFN-γ in controlling intracellular parasite infection [23]–[26]. However it is completely new for us that this cytokine is unique in controlling infection by *T. cruzi* G strain. It is generally observed that *T. cruzi* strains are sensible to most of immune response mechanisms and also that are able to overcome these responses establishing an acute phase characterized by parasitemia and animal death [6]–[9]. Indeed, this is the first report which showed that the traditionally well known immune response mechanism based on IFN-γ production is sufficient to control infection by low virulent *T. cruzi* G strain.

## Methods

### Animals

Female wild type BALB/c and C57BL/6 mice and also, iNOS, Nod2, Myd88, IL-12p40, TNF-α, IFN-γ, CD4, CD8, IL-18, gp91 phox subunit of NADPH oxidase knockout (KO) were provided and maintained at the animal facilities of the Department of Biochemistry and Immunology, School of Medicine of Ribeirão Preto, USP (Ribeirão Preto, Brazil). Male or female mice were six to eight weeks old and were kept under standard conditions on a 12-h light, 12-h dark cycle in a temperature-controlled room (25±2°C) with food and water *ad libitum* Maintenance and care of these animals complied with the guidelines of the Laboratory Animal Ethics Committee from the Institution. Animal euthanasia was performed in accordance with international welfare grounds, according to the American Veterinary Medical Association Guidelines on Euthanasia.

### Parasites and cells

*T. cruzi* from G was maintained in Vero cells culture. To obtain the amastigotes forms, trypomastigotes were incubated in LIT medium (liver infusion tryptose), pH 5.8 overnight. Vero, HeLa and MEF (murine embryonic cells) cells were maintained in Dulbecco's modified Eagle's medium (DMEM) (Gibco BRL, Gaithersburg, MD) with L-glutamine and Dglucose (4500 mg/L), sodium bicarbonate (2000 mg/L), HEPES (2380 mg/L), sodium pyruvate (1100 mg/L), supplemented with Fetal bovine serum (10%) and Penicillin (60 mg/L), gentamicin (40 mg/L) and streptomycin (10 mg/L). Cells were grown at 37°C with 5% CO_2_ in cell plates.

### *In vitro* multiplication assay

HeLa and MEF cells were plated into 24 wells plate (10^5^ cells/well). Each well contained a 13 mm round coverslips and were left overnight. After, amastigotes from G (20 parasites/cell) strain were put in contact with cells for 3 hours.After, wells were washed three times with PBS to remove non-internalized parasites. 3 and 48 hours post-infection cells were fixed with Bouin solution and Giemsa stained. Then, coverslips were glued onto glass slides. Number of internalized parasites and multiplication were counted in a total of 100 infected cells. The experiment was performed in triplicate and three times.

### *Ex-vivo* multiplication assay in inflammatory peritoneal macrophages

Inflammatory peritoneal macrophages, from C57BL/6, were recruited with the injection of thioglicollate 3% (3 g/L). Two days after, animals were intraperitoneally injected with 10^5^ amastigotes from G strain and macrophages extracted only after 3 hours. The cells were plated into 24 well plates (5×10^5^ cells/well). Finally, cells were Bouin fixed and Giemsa stained, 48 and 72 hours post-inoculation and number of internalized parasites was counted in a total of 100 infected macrophages. The experiment was performed in triplicate and three times.

### Release assay

Undifferentiated cells were extracted from C57BL/6 bone marrow. Primarily, the femur of mice was withdrawn and cells were extracted with a PBS squirt into the marrow. Afterwards, these cells were placed on a Petri dish with a medium containing 20% of fetal bovine serum and 30% of the supernatant of L929 cell line which secretes M-CSF (macrophage colony-stimulating factor) a macrophage differentiated factor. Once differentiated, cells were plated in a 96 well plate, some of them stimulated with 10 or 100 ng/mL of IFN-γ and others not. Subsequently, cells were infected with amastigotes from G strain (20 parasites/cell) and the release of trypomastigotes was observed over ten days.

### Parasitemia and mortality analysis

BALB/c, C57BL/6 and iNOS, TNF-α, IL-12p40, IL-18, CD4, CD8, IFN-γ and gp91 phox subunit of NADPH oxidase KO animals were intraperitoneally inoculated with 10^5^ amastigotes from G strain. Each group was composed of five animals. Blood was collected from animal orbital plexus and 5 µL was placed on a slide to parasitemia analyses. Parasitemia and animals mortality was observed over thirty days post-inoculation.

### Parasitemia and mortality analysis of inoculated and immunosuppressed mice

C57BL/6 mice were intraperitoneally inoculated with 10^5^ amastigotes from G strain. Each group was composed of five animals. Ten days after inoculation, the animals were immunosuppressed with Decadron (dexamethasone) 10 µg/mL. The medication was added to the water bottle of the immunossuppressed groups. Control groups were given just water. Blood was collected from animal tail and 5 µL was placed on a slide to parasitemia analyses. Parasitemia and animals mortality was observed over forty days post-inoculation.

### Flow cytometry

C57BL/6 mice were intraperitoneally inoculated with 10^5^ amastigotes from G strain. Each group was composed of four female animals. Control group was not infected. Blood was collected through orbital plexus at 8 and 25 days post-inoculation. Lymphocytes were separated from other blood cells using Ficoll-PaqueTM gradient (Amersham Biosciences).Cells were washed with FACS buffer, counted, and 5×10^5^cells were labeled with CD16/32-APC and CD69-PE or NK1.1-PE (BD). NK1.1 is a surface molecule expressed in NK cells in selected strains of mice, including C57BL/6 (an specific marker); CD16 and/or CD32 are expressed on NK, monocytes, macrophages, dendritic cells, kupffer cells, granulocytes, mast cells, B lymphocytes, immature thymocytes and some activated mature T lymphocytes (here an unspecific marker); CD69 is expressed upon activation of lymphocytes (T, B, NK, and NK-T cells), neutrophils and macrophages, also on IL-2 activated NK cells (an activation marker).The samples were acquired by FACSCantoII (BD), and the results were analyzed by FlowJo software (version 7.6.3).

### Statistical analyses

The significance of experiments was determined by one way ANOVA performed according to VassarStats program (Richard Lowry 1998–2006), available http://faculty.vassar.edu/lowry/VassarStats.html or by GraphPad Prism program, version 5.01 for Student-t analysis. The results were considered significant when p<0.05.The mortality analysis was performed by a survival curve according to GraphPad Prism program.

## Results

### Amastigotes from G strain provided a patent *in vitro* infection. On the other hand, was rapidly controlled *in vivo* but was not completely eliminated

**Figure 1:**
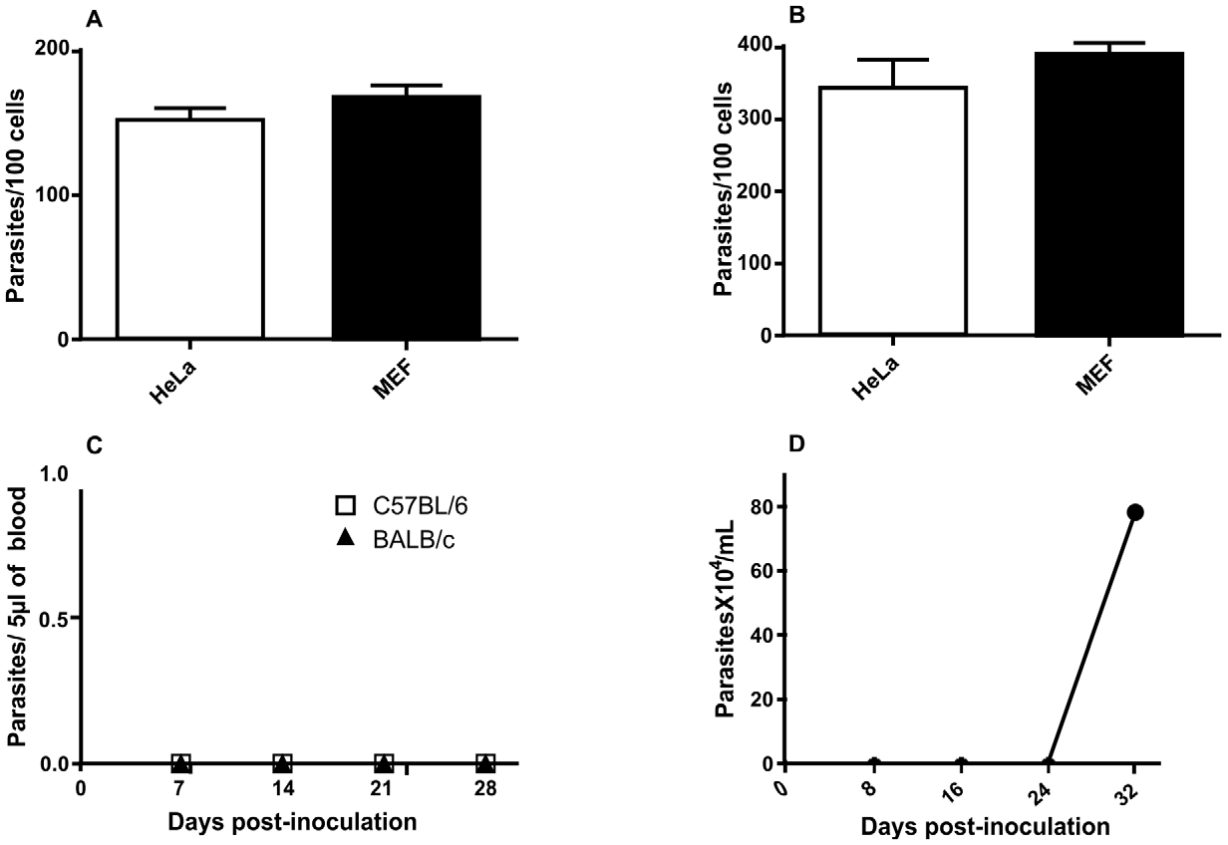
G strain parasitemia is only detected after dexmethasone treatment. G strain amastigote progeny is generated in Hela and MEF cells *in vitro* but no trypomastigotes are released at a detectable level in the bloodstream in C57BL/6 mice except if the latter are given dexmethasone. Amastigotes from G strain invasion (**A**) and multiplication (**B**) in HeLa and MEF cells. Parasitemia was not observed in wild type C57BL/6 and BALB/c mice (**C**). C57BL/6 mice that were given 100,000 amastigotes intraperitoneally at day 0 and that were given dexamethasone from day 10 onward displayed parasitemia from days 24 post amastigote inoculation (**D**). *p<0.001.

Invasion assays using amastigotes from G strain were performed during 3 hours and the multiplication verified 48 h post HeLa and MEF cells invasion. The results showed that G strain amastigotes showed high invasion and multiplication indexes in both mammalian cell lines (Figure 1 A and B). Also, 80 to 90% of cells were infected by the parasite. However, when BALB/c and C57BL/6 mice were intraperitoneally inoculated with amastigotes from G strain, no parasitemia was observed in both animal models (Figure 1 C). One could argue that this *T. cruzi* strain would be non-virulent. Nonetheless, in immunossupressed C57BL/6 animals, parasitemia reached high peak after 24 days post-inoculation (Figure 1 D).

### IFN-γ controls G strain amastigotes infection

**Figure 2:**
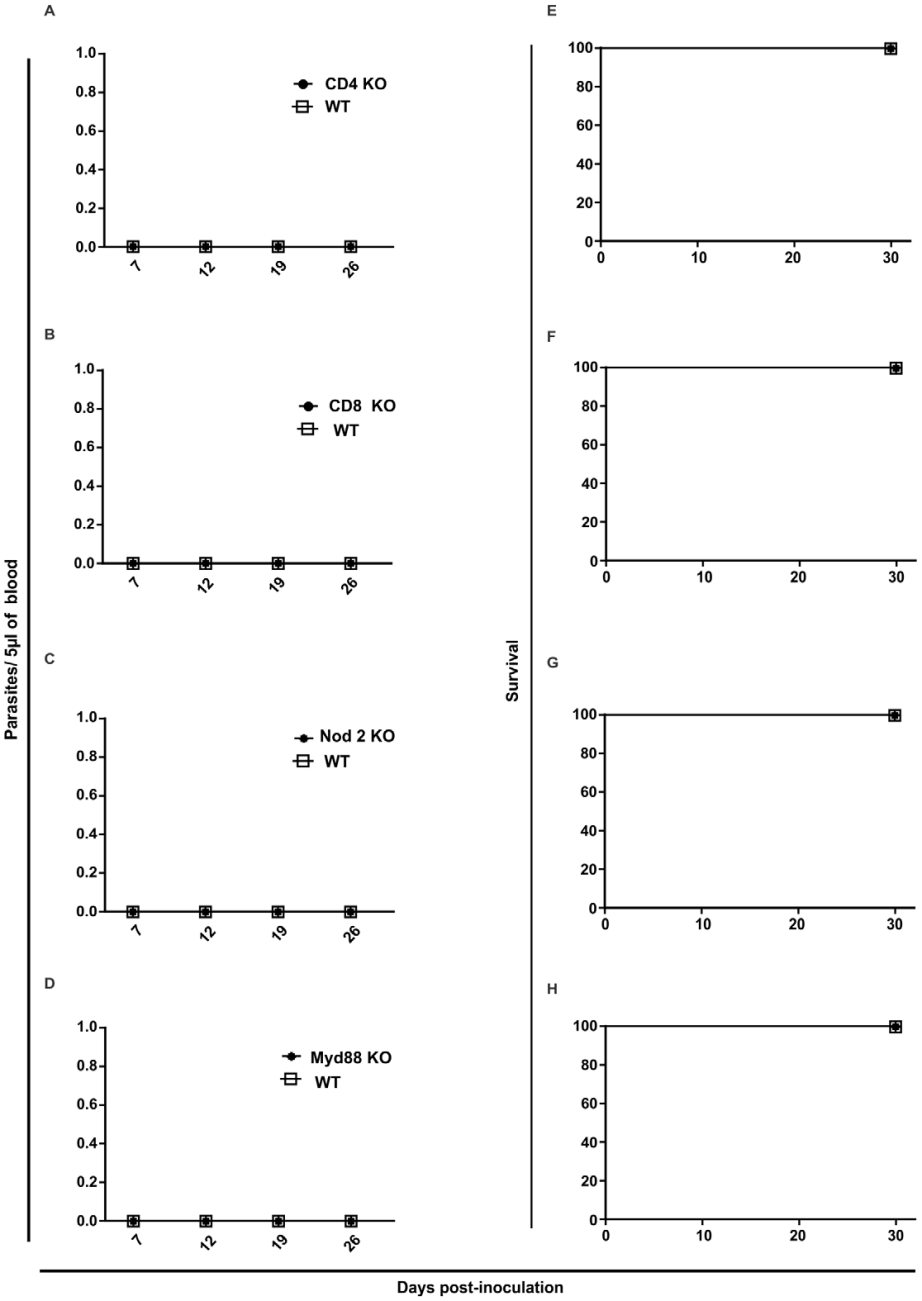
Whatever gene (CD4, CD8, Nod2 and Myd88) deletion, trypomastigotes were never detected in mice bloodstream. Wild type and CD4 (a, e), CD8 (b, f) Nod2 (c, g) and Myd88 (d, h) knockout mice mice were given intraperitoneally 100,000 G strain amastigotes. Parasitemia values were monitored in mouse blood at 7, 12, 19 and 26 days post-inoculation; survival was checked every day until 30 post-inoculation . (n = 5 mice per group).

**Figure 3:**
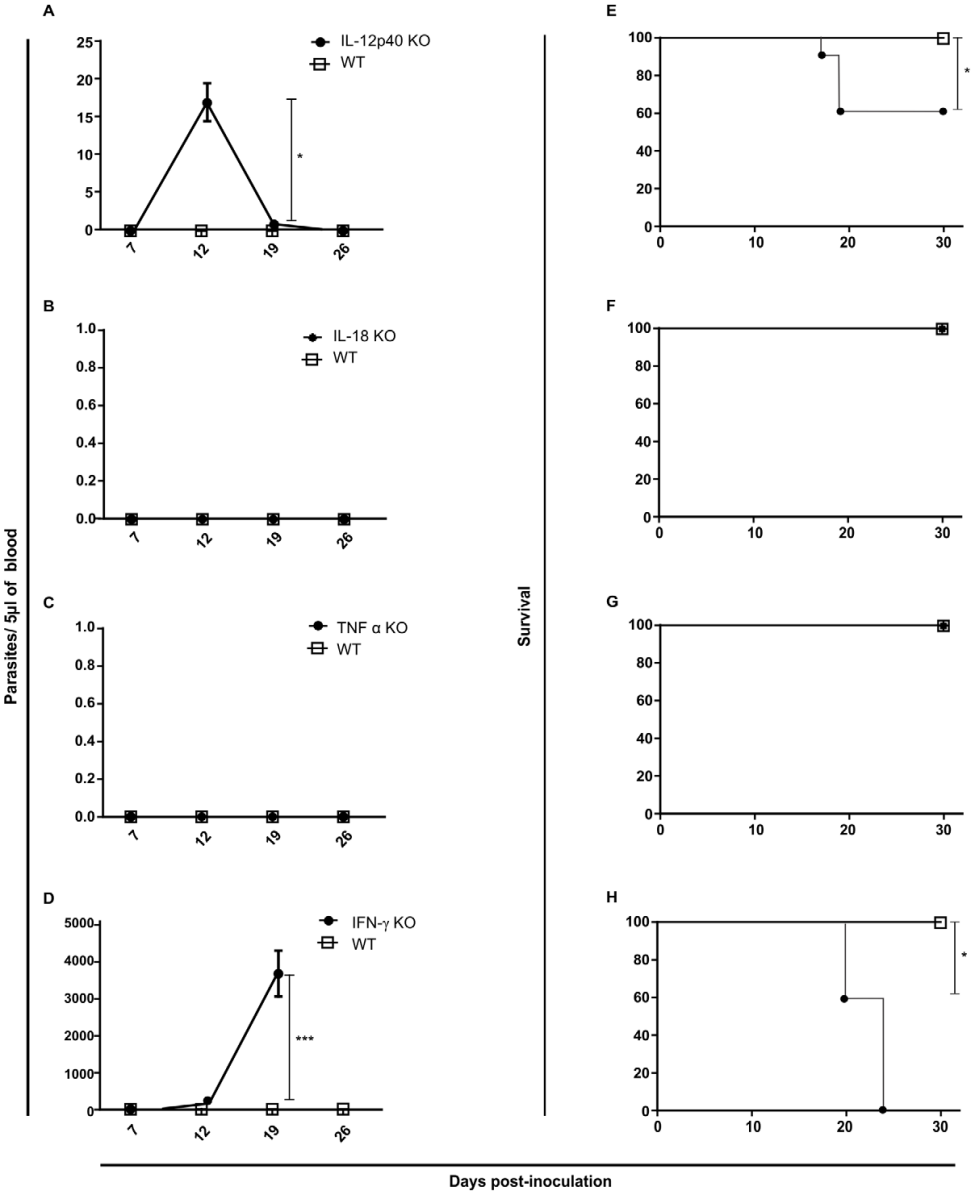
Deletion in IL12p40 and IFN-γ induced bloodstream parasitemia. Though with distinct profiles, in mice genetically deleted from either IL12p40, IFN-γ, the G strain trypomastigote progeny was detected in the bloodstream, while in mice deleted from either IL-18 or TNF-α, no trypomastigote progeny was detected. Wild type and IL-12p40 (a, e), IL-18 (b, f), TNF-α (c, g) and IFN-γ (d, h) knockout mice were given intraperitoneally 100,000 G strain amastigotes. Parasitemia values were monitored in mouse blood at 7, 12, 19 and 26 days post-inoculation; survival was checked every day until 30 post-inoculation . (n = 5 mice per group). It was observed parasitemia peak and mortality only for IL-12p40 and IFN-γ KO mice. *p<0.01; ***p<0.001.

**Figure 4:**
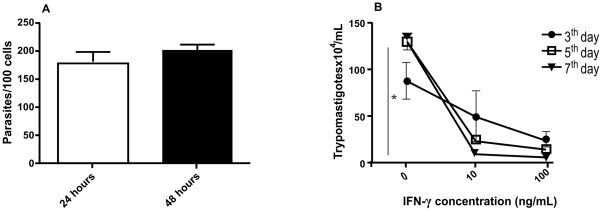
Inflamatory and IFN-γ treated naive macrophages impaired cell-cycling trypomastigotes differentiate from amastigotes. Amastigotes did not multiply in inflammatory peritoneal macrophages in an *ex-vivo* assay (**A**). Treatment with recombinant IFN-γ controlled in a dose dependent manner trypomastigotes release from bone marrow derived naive macrophages (**B**) (p<0.001).

**Figure 5:**
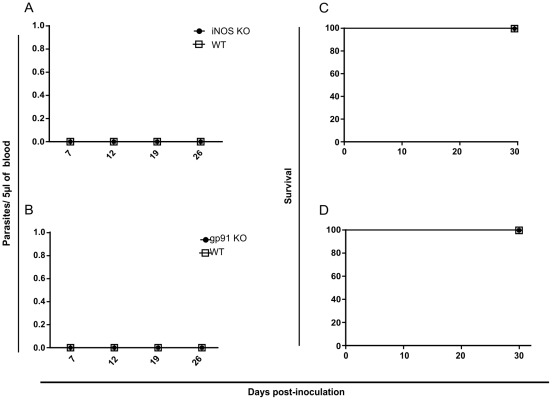
In iNOS and gp91 KO mice no trypomastigote progeny was detected. Wild type, iNOS (a, c) and gp91 KO (b, d) mice were given intraperitoneally 100 000 G strain amastigotes. Parasitemia values were monitored in mouse blood at 7, 12, 19 and 26 days post-inoculation ; survival was checked every day until 30 post-inoculation . (n = 5 mice per group).

In order to verify the impact of different host immune components in protection against infection by amastigotes from *T. cruzi* G strain we performed a screening using different knockout mice model. First, we inoculated Myd88, Nod2, CD4 and CD8 KO animals. The results showed no change in the course of infection and mortality comparing to WT mice (Figure 2 a, b, c, d, e, f, g, and h). After, we verified if cytokines would play any role in animal protection against amastigotes from *T. cruzi* G strain infection. In this sense, we used TNF-α, IL-18, IL-12 and IFN-γ KO mice. We observed that deficiency on TNF-α and IL-18 secretion did not have impact on parasitemia and animal survival (Figure 3 b, c, f and g). On the other hand, IL12p40 KO mice showed parasitemia on the 12 day post-inoculation (p<0.01) and 40% of mortality by the 30 post-inoculation (p<0.01) (Figure 3 a and e). Moreover, IFN-γ KO mice presented a high parasitemia peak by the 16 day post-inoculation (p<0.001) and all animals died by the 24 day (p<0.01) (Figure 3 d and h). These results turned our attention to the role of macrophages during *T. cruzi* G strain clearance. To gain insight about this issue, we performed an *ex vivo* assay. For that purpose, C57BL/6 mice were intraperitoneally inoculated with amastigotes from G strain for 3 h. Inflammatory peritoneal macrophages were collected and seeded into coverslips. After 48 and 72 h of culture the number of intracellular amastigotes was counted. It was observed that G strain amastigotes did not multiply intracellularly in the macrophage cultures (Figure 4 A). Moreover, naive macrophages obtained from C57BL/6 mice bone marrow undifferentiated cells were *in vitro* infected with amastigotes from G strain and the number of trypomastigotes in the supernatant was counted after three, five and seven days of infection and treatment or not with different concentrations of IFN-γ. The number of trypomastigotes from G strain in the supernatant was higher in non-treated cells (p<0.001), showing that naive macrophages could not impair parasite multiplication and differentiation. However, the number of released parasites was dramatically reduced in treated cells in an IFN-γ dose dependent manner (Figure 4 B). The next step was to identify the mechanism activated by macrophages during parasite clearance. For that purpose we inoculated amastigotes from G strain in iNOS and gp91 KO mice. However, the results showed no difference in the course of infection, neither on the mortality rates (Figure 5 a, b, c and d).

### NK cells are recruited early during infection by G strain amastigotes

**Figure 6:**
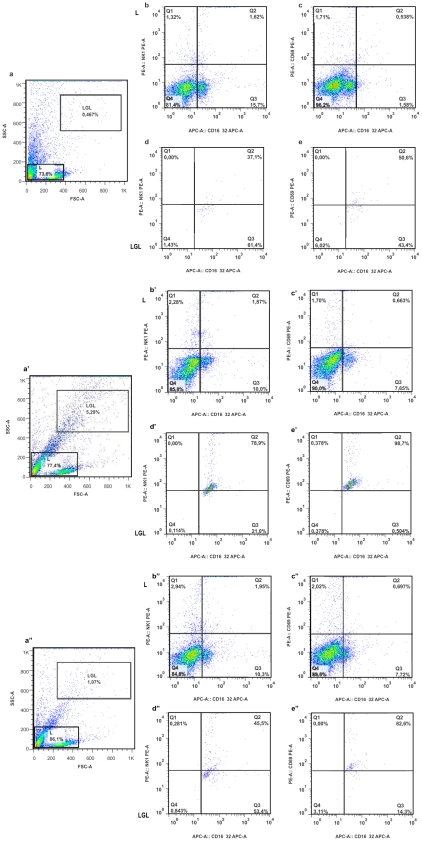
Monitoring presence of activated NK cells in bloodstream post amastigote intraperitoneal inoculation. Flow cytometry was performed with mononuclear cells prepared from mice left without any inoculation (a, b, c, d and e), and mice that were given intraperitoneal G strain amastiogotes at day-8 post-inoculation (a′, b′, c′, d′ and e′) at day-25 (b″, c″, d″ and e″). Note the higher percentage of activated NK cells at day-8 post-inoculation (p<0.01). Gates: L – lymphocytes and LGL – large granular lymphocytes (NK cells).

One important question raised by our results is the source of IFN-γ, since CD4 and CD8 cells seemed not to play an important role. Also, it is worth mentioning that the control occurred during the first days after inoculation. Thus, we performed flow cytometry in order to verify if NK cells were recruited during infection. The results showed that in non inoculated animals the lymphocytes population in peripheral blood were stained neither for CD16/32 nor for NK1.1 and CD69, indicating a phenotype of inactivated T cells (Figure 6 b and c). However, when we observed the animals by 8 days post-inoculation, we were capable of identifying another distinct cell population, which was denominated of “large granular lymphocytes” (LGL). It is know that the NK cells are largest than other lymphocytes and they have granular contents. Thereby, this LGL population had the same NK cells phenotype. We observed that this population was mostly double positive to CD16/32 and NK1.1 (Figure 6 d′) or CD69 (Figure 6 e′), confirming NK phenotype, and that they were activated. Afterward, our results demonstrated a dramatic increase in activated NK cell by the 8 day post-inoculation (p<0.001) (Figure 6 a′, d′, and e′), however this behavior was not maintained in 25 day post-inoculation, returning to basal levels (Figure 6 a″ d″ and e″).

## Discussion

*T. cruzi* is a very heterogeneous flagellate parasite; and its populations are characterized by a diverse morphology, a heterogeneous biological behavior, a high genetic variability, and distinctly different clinical courses. The clonal-histotropic model of Chagas' disease [27] describes a correlation between the clonal-population structure of *T. cruzi* and its tissue tropism; and it gives a possible explanation for the variety shown by this parasite. It is now accepted that *T. cruzi* strains can be divided into six DTUs, *T. cruzi* I to VI [13].

To our awareness this is the first study that evaluated the immune response against *T. cruzi* amastigotes from strain belonging to group I. In this context, understanding the way host responds to amastigotes is quite important, once amastigotes are the main forms encountered during the chronic phase of the disease. Our first observation is that infection by amastigotes from G strain did not activate signal pathway dependent on Myd88 nor reliant on Nod2 receptor. Probably other innate immune response related receptors are triggered during amastigotes infection. This issue will be addressed in additional studies. Infection in other knockout animals showed that G strain amastigotes were only susceptible to IL-12 and IFN-γ production. The major cytokine responsible for IFN-γ secretion is IL-12. However we observed just a low peak of parasitemia in IL-12 KO mice. This result may be explained by the fact that IL-18 also induces IFN-γ secretion. Moreover, IL-18 KO mice showed no parasitemia. Thus, these results showed the redundant role of these cytokines in inducing IFN-γ production and infection control. A double knockout mice model for both cytokines would be helpful to sustain this hypothesis.

IFN-γ is an important mediator of resistance to *T. cruzi*. Besides iNOS, IFN-γ regulates the expression of a large number of genes, including chemokines and chemokine receptors, which were shown to play a role in IFN-γ mediated protection in *T. cruzi* infection. Early during infection, IFN-γ is secreted by NK cells and other cell types, as part of the innate response, and later on the infection course by activated CD4+ and CD8+ T cells [23]–[25]. Recently, authors demonstrated for the first time *in vivo*, the specific importance of direct IFN-γ mediated activation of macrophages for controlling infection with multiple protozoan parasites [26]. Here we observed that IFN-γ plays crucial and unique role in controlling infection by amastigotes from *T. cruzi* G strain.

Nitric oxide (NO) and reactive oxygen species (ROS) are two key inflammatory mediators involved not only in pathogen clearance but also in tissue injury. Nitric oxide is produced by different isoforms of NO synthase, among them the inducible isoform (iNOS) that is activated by IFN-γ and TNF-α [28]. During *T. cruzi* infection, NO can directly or indirectly modulate the effector leukocyte machinery through diverse mechanisms. This process involves microbicidal effects derived from toxic-free radicals (peroxinitrite and superoxide) generated after NO production, as well as regulation/enhancement of the inflammatory response induced during this type of infection, a dual role in the immunity that is usually observed for NO. This well-known immune duality is usually dependent on concentration and, once dysregulated, may lead to host cell toxicity, autoimmunity or parasite persistence due to immune evasion, all of which can lead to pathology. NO is involved in the control of *T. cruzi*-induced parasitemia and directly kills the parasite *in vitro*. NO affects *T. cruzi* by chemically modifying cysteine-containing proteins and/or by binding to metallo-proteins that mediate crucial metabolic processes. The strength of NO toxicity is dependent on the sensitivity of the parasite, which differs among parasite strains and according to the physiological microenvironment [29]. Moreover, Oxidative burst of activated phagocytes results in the release of ROS, e.g., superoxide (O_2_^•−^), hydrogen peroxide (H_2_O_2_), and hydroxyl radical, via activation of NADPH oxidase (NOX) and/or myeloperoxidase (MPO) enzymes. The inflammatory cytokines and ROS are important for the control of *T. cruzi* and may be cytotoxic to the host cellular components. Many of the ROS are highly reactive and diffusible and may be released into the extracellular milieu. Whereas intracellular ROS serve mainly for host defense against infectious agents, the extracellular release of ROS, when present in abundance, directly damages the surrounding tissues or promotes inflammatory processes (revised in [30]). Our results obtained from iNOS and gp91 KO mice showed no parasitemia during the 30 days post-inoculation. Thus, is conceivable to believe that NO and ROS may play redundant role during parasite clearance. Another hypothesis that does not completely exclude the first is that other mechanisms of parasite clearance would be activated by G strain parasites, such as a group of IFN-γ induced genes that plays a major role in host control of intracellular pathogens. These genes belong to a family encoding a series of 47- to 48-kDa GTPases for instance LRG-47 that can influence *T. cruzi* Y strain control by simultaneously regulating macrophage-microbicidal activity and hemopoietic function [31].

Our results showed the impact of innate immune response in controlling infection by amastigotes from G strain. In addition, CD4 and CD8 KO mice showed no difference in the infection course. This information may represent important finding to design novel immune strategies focused on enhancing the innate immune response to control pathology that may be caused by different strains of the parasite in the same host.

To gain insight into the source of IFN-γ production, we performed flow cytometry and observed that the lymphocyte population in the peripheral blood samples showed an inactivated phenotype in infected and non infected animals. While in infected animals, we observed a significant increase in NK population with an activated phenotype. This result suggested that the main source of IFN-γ produced to protect animal against amastigotes from *T. cruzi* G strain is NK cells. However, depletion of NK cell in WT and IFN-γ KO mice would be interesting to confirm the hypothesis.

In conclusion, our research showed that although amastigotes from G strain were highly infective *in vitro* they did not induce a patent infection *in vivo* due to the high susceptibility to IFN-γ production early in infection. This study highlighted the need to consider strain biases when investigating host immune response against *T. cruzi*.

## References

[1] Rassi A, Rassi A, Marin-Neto JA (2010). Chagas disease.. The Lancet.

[2] Brener Z, Gazzinelli RT (1997). Immunological control of *Trypanosoma cruzi* infection and pathogenesis of Chagas' disease.. Int Arch Allergy Immunol.

[3] Dos Reis GA (1997). Cell-mediated immunity in experimental *Trypanosoma cruzi* infection.. Parasitol Today.

[4] Kayama H, Takeda K (2010). The innate immune response to *Trypanosoma cruzi* infection.. Microbes Infect.

[5] Dos Reis GA (2011). Evasion of immune responses by *Trypanosoma cruzi*, the etiological agent of Chagas disease.. Braz J Med Biol Res.

[6] Aliberti JCS, Cardoso MAG, Martins GA, Gazzinelli RT, Vieira LQ (1996). Interleukin- 12 mediates resistance to *Trypanosoma cruzi* in mice andis produced by murine macrophages in response to live trypomastigotes.. Infection and Immunity.

[7] Aliberti JCS, Machado FS, Souto JT, Campanelli AP, Teixeira MM (1999). *β*- Chemokines enhance parasite uptake and promote nitricoxide-dependent microbiostatic activity in murine inflammatory macrophages infected with *Trypanosoma cruzi*.. Infection and Immunity.

[8] Antúnez MI, Cardoni RL (2000). IL-12 and IFN-*γ* production, and NK cell activity, in acute and chronic experimental *Trypanosoma cruzi* infections.. Immunology Letters.

[9] Talvani A, Ribeiro CS, Aliberti JC, Michailowsky V, Santos PV (2000). Kinetics of cytokine gene expression in experimental chagasic cardiomyopathy: tissue parasitism and endogenous IFN-*γ* as important determinants of chemokine mRNA expression during infection with *Trypanosoma cruzi*.. Microbes and Infection.

[10] Abrahamsohn IA, Coffman RL (1996). *Trypanosoma cruzi*: IL-10, TNF, IFN-*γ* and IL-12 regulate innate and acquired immunity to infection.. Experimental Parasitology.

[11] Rodrigues MM, Ribeirão M, Boscardin SB (2000). CD4 Th1 but not Th2 clones efficiently activate macrophages to eliminate *Trypanosoma cruzi* through a nitric oxide dependent mechanism.. Immunology Letters.

[12] Hiyama K, Hamano S, Nakamura T, Nomoto K, Tada I (2001). IL-4 reduces resistance of mice to *Trypanosoma cruzi* infection.. Parasitology Research.

[13] Zingales B, Andrade SG, Briones MR, Campbell DA, Chiari E (2009). A new consensus for *Trypanosoma cruzi* intraspecific nomenclature: second revision meeting recommends *T. cruzi* I to TcVI.. Mem Inst Oswaldo Cruz.

[14] Di Noia JM, Buscaglia CA, De Marchi CR, Almeida IC, Frasch ACC (2002). A *Trypanosoma cruzi* small surface molecule provides the first immunological evidence that Chagas' disease is due to a single parasite lineage.. Journal of Experimental Medicine.

[15] Fernandes O, Mangia RH, Lisboa CV, Pinho AP, Morel CM (1999). The complexity of the sylvatic cycle of *Trypanosoma cruzi* in Rio de Janeiro state (Brazil) revealed by the non-transcribed spacer of themini-exon gene.. Parasitology.

[16] Añez N, Crisante G, da Silva FM, Rojas A, Carrasco H (2004). Predominance of lineage I among *Trypanosoma cruzi* isolates from Venezuelan patients with different clinical profiles of acute Chagas' disease.. Tropical Medicine and International Health.

[17] Black CL, Ocaña S, Riner D, Costales JA, Lascano MS (2007). Household risk factors for *Trypanosoma cruzi* seropositivity in two geographic regions of Ecuador.. Journal of Parasitology.

[18] Mejía-Jaramillo AM, Peña VH, Triana-Chávez O (2009). *Trypanosoma cruzi*: biological characterization of lineages I and II supports the predominance of lineage I in Colombia.. Experimental Parasitology.

[19] Yoshida N (1983). Surface antigens of metacyclic trypomastigotes of *Trypanosoma cruzi*.. Infection and Immunity.

[20] Yoshida N, Blanco SA, Araguth MF, Russo M, González J (1990). The stage-specific 90-Kilodalton surface antigen of metacyclic trypomastigotes of *Trypanosoma cruzi*.. Mol Biochem Parasitol.

[21] Silva CV, Luquetti AO, Rassi A, Mortara RA (2006). Involvement of Ssp-4-related carbohydrate epitopes in mammalian cell invasion by *Trypanosoma cruzi* amastigotes.. Microbes and Infection.

[22] Silva CV, Kawashita SY, Probst CM, Dallagiovanna B, Cruz MC (2009). Characterization of a 21 kDa protein from *Trypanosoma cruzi* associated with mammalian cell invasion.. Microbes and Infection.

[23] Gazzinelli RT, Oswald IP, Hieny S, James SL, Sher A (1992). The microbicidal activity of interferon-gamma-treated macrophages against *Trypanosoma cruzi* involves an L-arginine-dependent, nitrogen oxide-mediated mechanism inhibitable by interleukin-10 and transforming growth factor-beta.. Eur J Immunol.

[24] Hölscher C, Köhler G, Müller U, Mossmann H, Schaub GA (1998). Defective nitric oxide effector functions lead to extreme susceptibility of *Trypanosoma cruzi*-infected mice deficient in gamma interferon receptor or inducible nitric oxide synthase.. Infect Immun.

[25] Aliberti JC, Souto JT, Marino AP, Lannes-Vieira J, Teixeira MM (2001). Modulation of chemokine production and inflammatory responses in interferongamma- and tumor necrosis factor-R1-deficient mice during *Trypanosoma cruzi* infection.. Am J Pathol.

[26] Lykens JE, Terrell CE, Zoller EE, Divanovic S, Trompette A (2010). Mice with a selective impairment of IFN-gamma signaling in macrophage lineage cells demonstrate the critical role of IFN-gamma-activated macrophages for the control of protozoan parasitic infections in vivo.. J Immunol.

[27] Padilla AM, Bustamante JM, Tarleton RL (2009). CD8+ T cells in *Trypanosoma cruzi* infection.. Curr Opin Immunol.

[28] Bogdan C (2001). Nitric oxide and the immune response.. Nat Immunol.

[29] Gutierrez FRS, Mineo TWP, Pavanelli WR, Guedes PMM, Silva JS (2009). The effects of nitric oxide on the immune system during *Trypanosoma cruzi* infection.. Mem Inst Oswaldo Cruz.

[30] Gupta S, Dhiman M, Wen JJ, Garg NJ (2011). ROS signalling of inflammatory cytokines during *Trypanosoma cruzi* infection.. Adv Parasitol.

[31] Santiago HC, Feng CG, Bafica A, Roffe E, Arantes RM (2005). Mice Deficient in LRG-47 Display Enhanced Susceptibility to *Trypanosoma cruzi* Infection Associated with Defective Hemopoiesis and Intracellular Control of Parasite Growth.. J Immunol.

